# Dexamethasone stimulates expression of C-type Natriuretic Peptide in chondrocytes

**DOI:** 10.1186/1471-2474-7-87

**Published:** 2006-11-20

**Authors:** Hanga Agoston, Laurie Baybayan, Frank Beier

**Affiliations:** 1 CIHR Group in Skeletal Development and Remodeling, Department of Physiology and Pharmacology, University of Western Ontario, London, ON, Canada

## Abstract

**Background:**

Growth of endochondral bones is regulated through the activity of cartilaginous growth plates. Disruption of the physiological patterns of chondrocyte proliferation and differentiation – such as in endocrine disorders or in many different genetic diseases (e.g. chondrodysplasias) – generally results in dwarfism and skeletal defects. For example, glucocorticoid administration in children inhibits endochondral bone growth, but the molecular targets of these hormones in chondrocytes remain largely unknown. In contrast, recent studies have shown that C-type Natriuretic Peptide (CNP) is an important anabolic regulator of cartilage growth, and loss-of-function mutations in the human CNP receptor gene cause dwarfism. We asked whether glucocorticoids could exert their activities by interfering with the expression of CNP or its downstream signaling components.

**Methods:**

Primary mouse chondrocytes in monolayer where incubated with the synthetic glucocorticoid Dexamethasone (DEX) for 12 to 72 hours. Cell numbers were determined by counting, and real-time PCR was performed to examine regulation of genes in the CNP signaling pathway by DEX.

**Results:**

We show that DEX does influence expression of key genes in the CNP pathway. Most importantly, DEX significantly increases RNA expression of the gene encoding CNP itself (*Nppc*). In addition, DEX stimulates expression of *Prkg2 *(encoding cGMP-dependent protein kinase II) and *Npr3 *(natriuretic peptide decoy receptor) genes. Conversely, DEX was found to down-regulate the expression of the gene encoding its receptor, *Nr3c1 *(glucocorticoid receptor), as well as the *Npr2 *gene (encoding the CNP receptor).

**Conclusion:**

Our data suggest that the growth-suppressive activities of DEX are not due to blockade of CNP signaling. This study reveals a novel, unanticipated relationship between glucocorticoid and CNP signaling and provides the first evidence that CNP expression in chondrocytes is regulated by endocrine factors.

## Background

Bone formation involves the distinct, but related processes of intramembranous ossification and endochondral ossification [1,2]. While the former forms flatter bones like those of the skull, endochondral ossification is responsible for development of the long bones of the limbs, the vertebrae and the ribs. Endochondral ossification begins when mesenchymal cells condense, differentiate into chondroblasts and then proceed successively through the resting, proliferating, and hypertrophic chondrocyte stages in the cartilage growth plate [2,3]. The differentiation of mesenchymal cells into chondroblasts is regulated by the activity of the Sox9 transcription factor, which controls the expression of principal genes encoding the extracellular matrix proteins of cartilage, such as collagen type II and aggrecan [4]. Another transcription factor, Runx2, promotes hypertrophic differentiation and stimulates expression of type X collagen, a marker of hypertrophic chondrocytes [5]. The cartilage anlagen serve as the models of future bones, and the rate of proliferation and in particular the volume increase during chondrocyte hypertrophy are the driving forces for bone elongation that determine our final height. Due to the complex nature of cartilage development, it is critical to understand each step involved in regulation of this process, as there are many acquired and inherited cartilage diseases resulting from disturbances in this pathway, including glucocorticoid-induced growth retardation and human chondrodysplasias [6-8]. Recent studies have demonstrated an intricate weave of signaling pathways regulating endochondral ossification, including many hormones and growth factors, such as glucocorticoids and C-type natriuretic peptide (CNP) [6-9].

Long-term administration of anti-inflammatory glucocorticoids (for example in the treatment of childhood asthma, autoimmune diseases or pediatric cancers) results in growth retardation, bone loss, and possible premature or exaggerated osteoporosis [10]. Most glucocorticoid effects on endochondral bone growth appear to be due to direct regulation of chondrocytes, as opposed to generalized endocrine effects [11,12]. While effects of glucocorticoids on chondrocyte proliferation, differentiation and apoptosis as well as on vascular invasion of hypertrophic cartilage have been reported, the contributions of these effects to growth retardation and the molecular mechanisms involved are not completely understood [7,8,13]. Glucocorticoids signal largely through the glucocorticoid receptor (encoded by the *Nr3c1 *gene), a member of the nuclear receptor family that translocates into the nucleus upon ligand binding and acts as transcription factor [14], but the molecular targets of glucocorticoids in chondrocytes are largely unknown. Here, we investigated whether expression of genes involved in the CNP signaling pathway is impacted by the administration of a synthetic glucocorticoid, dexamethasone (DEX).

CNP is a member of the natriuretic peptide family consisting of atrial natriuretic peptide (ANP), brain/B-type natriuretic peptide (BNP) and CNP [15]. ANP and BNP act through the same membrane-bound guanylyl cyclase receptor GC-A or NPR1 (gene name in mouse: *Npr1*), while CNP acts through GC-B/NPR2 (*Npr2*) to initiate the cGMP-signaling cascade [9]. Elevation in intracellular cGMP levels in response to receptor-ligand interactions results in the activation of downstream mediators such as cyclic nucleotide phosphodiesterases (PDEs), cGMP-regulated ion channels, and cGMP-dependent protein kinases cGKI and cGKII (*Prkg1, Prkg2*, respectively) [9,16]. A third type of natriuretic peptide receptor (*Npr3*) does not posses guanylyl cyclase activity and is thought to act as a decoy/clearance receptor that regulates the levels of natriuretic peptides available for interaction with NPR1 and NPR2 [9].

Both ANP and BNP are primarily involved in body fluid regulation and cardiovascular function, while a range of functions have been described for CNP, including dilation of smooth muscle cells and regulation of endochondral bone growth [17,18]. CNP-deficient mice display dwarfism, with significantly reduced lengths of endochondral bones such as femur, tibia, and vertebrae [19]. Histological studies in these mice show decreased growth plate width, resulting from reduced proliferative and hypertrophic zones [19]. A similar phenotype was observed in mice deficient for the CNP receptor *Npr2 *[20,21]. Most notably, loss-of-function mutations in the human *NPR2 *gene cause reduced height and skeletal effects in acromesomelic dysplasia, type Maroteaux [22,23]. Conversely, CNP treatment results in enhanced endochondral bone growth in organ culture [24,25]. Moreover, ectopic CNP can rescue the effects of activating mutations in the gene encoding fibroblast growth factor receptor 3 in rodent models of achondroplasia, the most common form of human dwarfism [26,27]. In summary, there is strong evidence for both an obligatory role of endogenous CNP signaling in normal cartilage development and a potential therapeutic role for exogenous CNP in the treatment of skeletal growth disorders.

CNP is expressed by chondrocytes and appears to control endochondral ossification in an autocrine/paracrine manner [19], but the mechanisms regulating CNP expression in cartilage are unknown. In this study we asked whether glucocorticoids exert their growth-suppressing effects on bone growth by down-regulation of CNP signaling components.

## Methods

### Cell Culture and Cell Counts

All media components were purchased from Invitrogen, unless stated otherwise. Tibias from CD1 timed-pregnant mice (Charles River Canada) were isolated from 15.5 day embryos under a Stemi DV4 Stereomicroscope (Zeiss). Limb bones were allowed to recover from dissection overnight in serum-free organ culture media containing 0.2% Bovine Serum Albumin (BSA) (Fisher Scientific), 0.5 mM L-glutamine, 40 units penicillin/ml and 40 μg streptomycin/ml. The following day bones were digested with 0.25% trypsin/EDTA for 15 minutes, followed by digestion with 3 mg/mL of Collagenase-P (Roche) in DMEM and 10% FBS for 2 hours at 37°C with constant rotary motion. Cells were plated at a density of 500,000 cells/well in NUNC 6-well plates to adhere overnight in primary cell culture media, consisting of 60% F-12, 40%DMEM, 10% Fetal Bovine Serum (FBS), 0.5 mM L-glutamine, 40 units penicillin/ml and 40 μg streptomycin/ml. Monolayer cultures were treated with DMSO or DEX (10^-7^M) in DMSO vehicle the following morning. Cells were harvested before treatment and at 12, 24, 48, and 72 hr time points. RNA from primary cells was isolated using QIAGEN (Mississauga) RNeasy^® ^Mini kit and protocol for animal cells.

For cell counts, 100,000 chondrocytes per well were plated into 24-well plates in primary cell culture medium supplemented with DMSO, 10^-6 ^or 10^-7 ^M DEX. After 72 hours, cells were counted using a hematocytometer. Cell counts present average and standard deviation from three independent experiments, performed in triplicate each.

### Real-Time PCR

Real-Time PCR analysis for *Col2a1 *and *Col10a1 *were performed as described [28-30] using the Applied Biosystems 7900HT Real-Time PCR System and TaqMan^® ^Gene Expression Assays. *Nppc, Npr2, Npr3, Nr3c1, Prkg1, Prkg2 *and *Gapdh *probes were purchased as Assays-on-demand (Applied Biosystems) and used the same ways as the *Col2a1 *and *Col10a1 *probes. Gene expression levels were determined using the Standard Curve quantitative method with *Gapdh *levels as the basis of comparison. All data represent averages and SEM from three to four independent cell isolations.

### Statistical Analyses

Two-Way ANOVA (parametric) test with Bonferroni post-test was performed using the Graph Pad/Prism software.

## Results

**Figure 1:**
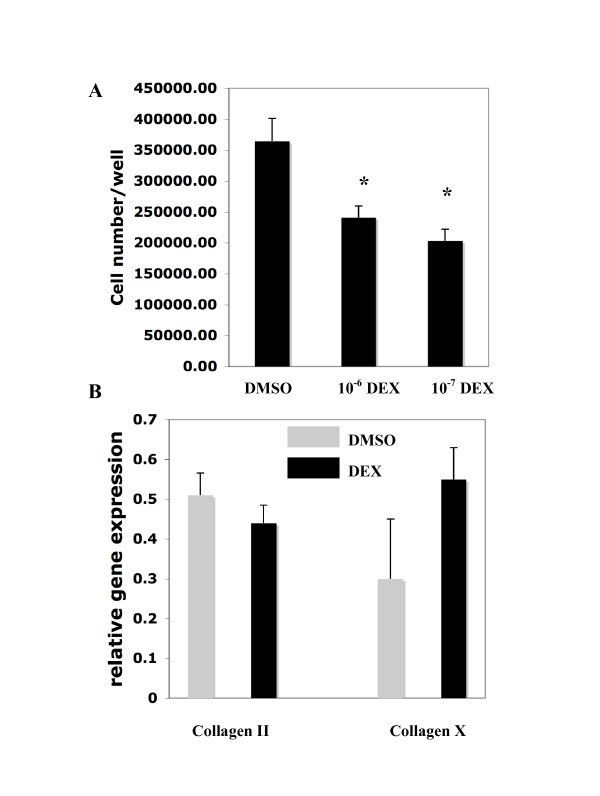
**DEX treatment reduces chondrocyte cell numbers**. A) Primary mouse chondrocytes were plated at a density of 100,000 cells/well, incubated with DMSO or 10^-6 ^or 10^-7 ^M DEX for 72 hours and counted. While cell numbers in control conditions (DMSO) increased 3.5-fold over the culture period, DEX treatment at both concentrations significantly reduced this increase. Data present averages and standard deviations from three independent experiments, performed in triplicate each (*P < 0.05). B) Primary chondrocytes were incubated with DMSO or 10^-7 ^M DEX for 72 hours, and mRNA levels for collagen II and collagen X genes were analyzed by real-time PCR. Neither gene was affected significantly by DEX. Data represent mean ± SEM from three independent trials.

We first examined whether DEX would affect the cell number of primary mouse chondrocytes in monolayer culture. In control cultures, cell numbers increased 3.6-fold over a 72 hour time course (Fig. 1A). In contrast, treatment with 10^-6 ^M DEX reduced this increase to 2.4-fold, in agreement with earlier studies [31-33]. 10^-7 ^M DEX caused an even larger reduction, allowing only a doubling of cell numbers. However, the differences between the two DEX concentrations were not statistically significant. Real-time PCR analyses at the same time point demonstrated that 10^-7 ^M DEX does not change the mRNA levels for collagen II significantly (Fig. 1B). DEX treatment appears to increase collagen X mRNA levels slightly, but this increase is not statistically significant (Fig. 1B). These data suggest that DEX treatment did not alter the differentiation status of chondrocytes in these experiments.

**Figure 2:**
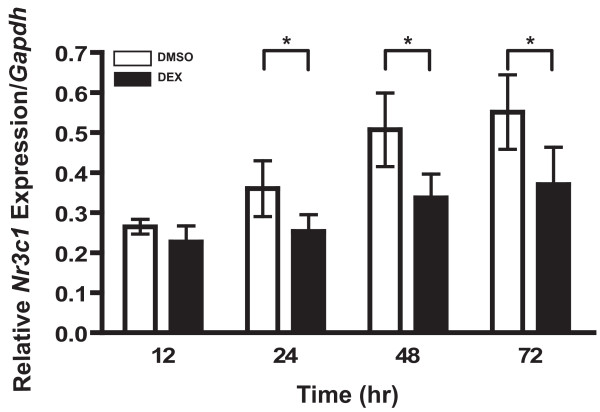
**DEX treatments results in down-regulation of *Nr3c1 *expression**. Primary chondrocytes were incubated with DMSO or 10^-7 ^M DEX for 12 to 72 hours, and *Nr3c1 *mRNA levels were determined by real-time PCR relative to *Gapdh*. *Nr3c1 *expression increased under control conditions during the time-course, but decreased upon DEX treatment. Data represent mean ± SEM from three independent trials (*P < 0.05).

We next asked whether DEX would regulate the expression of its own receptor. The expression of the glucocorticoid receptor gene (*Nr3c1*) was slightly upregulated after 48 and 72 hours in control cultures (Fig 2). However, beginning at the 24 hour time point, DEX significantly down-regulated glucocorticoid receptor mRNA expression. This effect was maintained throughout the time course, reaching maximal inhibition of 38% at 48 hours of DEX treatment.

**Figure 3:**
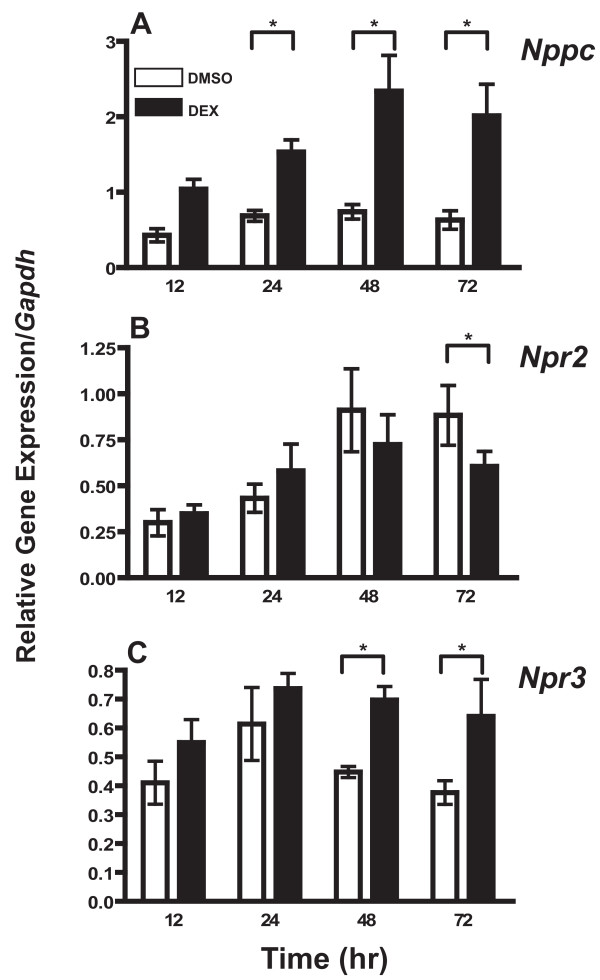
**DEX regulates expression of CNP and CNP receptors**. Primary chondrocytes were incubated with DMSO or 10^-7 ^M DEX for 12 to 72 hours, and *Nppc *(encoding CNP, A), *Npr2 *(encoding the CNP signaling receptor, B) and *Npr3 *(encoding the CNP decoy receptor, C) mRNA levels were determined by real-time PCR relative to *Gapdh*. *Nppc *expression is significantly increased upon DEX treatment, while DEX down-regulated *Npr2 *slightly and up-regulated *Npr3 *at the 48 and 72 hr time points. Data represent mean ± SEM from three or four independent trials (*P < 0.05).

Since DEX reduces chondrocyte cell numbers and CNP has shown to increase chondrocyte proliferation [19,24,25], we asked whether DEX treatment alters the expression of CNP or its receptors. In control conditions, the CNP gene (*Nppc*) was expressed at constant levels throughout the culture period (Fig. 3A). Surprisingly, DEX caused a significant increase in *Nppc *mRNA levels, starting at 12 hours and reaching 3.7-fold stimulation after 72 hours of treatment. Expression of the CNP receptor *Npr2 *increased almost three-fold over the time course under control conditions (Fig. 3B). DEX caused a slight, but significant reduction in *Npr2 *mRNA levels at 72 hours, with no effects at earlier time points. In control cells, expression of the decoy receptor *Npr3 *increased more rapidly that that of *Npr2*, reaching a three-fold increase after 24 hours and dropping slightly after that (Fig. 3C). DEX treatment counteracted this down-regulation and caused increased *Npr3 *mRNA levels at 48 and 72 hours.

**Figure 4:**
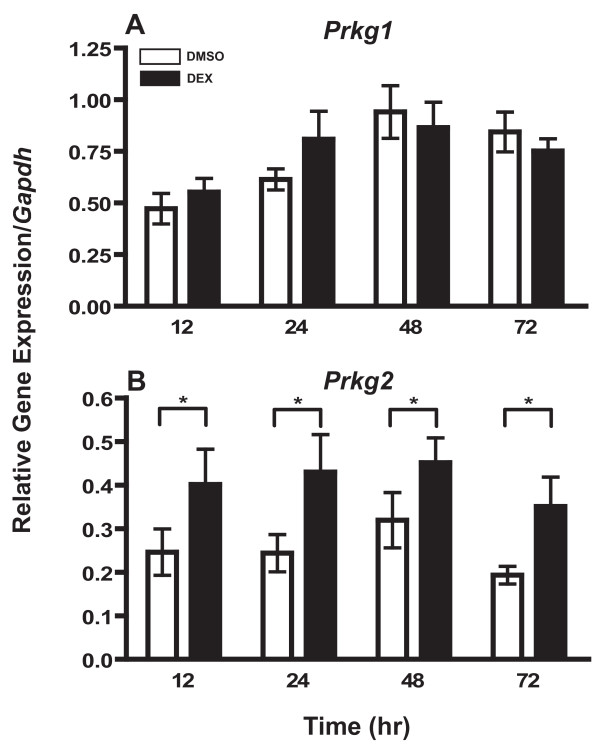
**DEX increases *Prkg2 *expression in chondrocytes**. Primary chondrocytes were incubated with DMSO or 10^-7 ^M DEX for 12 to 72 hours, and *Prgk1 *(encoding cGMP-dependent protein kinase I, A) and *Prkg2 *(encoding cGMP-dependent protein kinase II, B) mRNA levels were determined by real-time PCR relative to *Gapdh*. While *Prkg1 *expression remained relatively unchanged over time and in response to DEX treatment, *Prkg2 *levels were significantly increased during the time course and in response to DEX. Data represent mean ± SEM from three or four independent trials (*P < 0.05).

Finally, we examined the effects of DEX treatment on the expression of cGMP-dependent kinases I and II (*Prgk1 *and *Prkg2*), two of the main mediators of CNP signaling. *Prkg1 *gene expression increased slightly during the time course and was not affected by DEX treatment (Fig. 4A). Expression of *Prkg2 *remained relatively constant throughout the incubation period and was increased slightly, but significantly by DEX treatment (Fig. 4B). Similar to *Nppc *(but in contrast to the other genes examined), *Prkg2 *expression responded to DEX at the earliest time point investigated (12 hours).

## Discussion

The process of endochondral ossification involves finely controlled pathways that are not completely understood. These signalling networks likely include crosstalk between different pathways. Here, we have investigated how glucocorticoids impact the expression of various genes in the CNP cascade. It is known that sustained glucocorticoid treatment negatively impacts bone growth in humans, while CNP is a positive regulator of endochondral bone growth. Thus, we speculated that interaction of these two pathways, in particular down-regulation of endogenous CNP signaling by glucocorticoids, could account for some of the effects of these mediators.

We investigated this possibility using primary chondrocytes isolated from 15.5d ay old mouse embryonic forelimbs. Isolated cells were cultured with and without dexamethasone (DEX) for three days. DEX treatment partially inhibited the increase in cell numbers over the incubation period that was seen in control cultures [31-33]. Our data as well as previous studies suggest that this decrease most likely arises from reduced rates of proliferation, but a contribution from increased apoptosis cannot be excluded [31-37]. However, DEX treatment did not result in significant changes in either collagen II or collagen X mRNA expression, indicating that the differentiation status of chondrocytes is not markedly affected by DEX, at least within culture conditions applied here.

Because of the opposing effects of DEX and CNP on chondrocyte cell numbers, we asked whether DEX affects the expression of components of the CNP signalling system, using real-time PCR analyses. The most striking result of our studies was the almost four-fold induction of CNP mRNA expression by DEX. Since CNP and glucocorticoids have opposing effects on endochondral bone growth, these effects were unexpected. Down-regulation of mRNA for the CNP signaling receptor *Npr2 *and up-regulation of transcript levels for the decoy receptor *Npr3 *by DEX might counteract increased CNP levels to some degree; however, changes in the mRNA levels for the receptor genes are relatively minor compared to changes in CNP mRNA expression. Furthermore, DEX also increased expression of *Prkg2 *mRNA, an essential component of CNP signalling in endochondral bone growth [38]. Thus, it is unlikely that glucocorticoid-induced retardation of endochondral bone growth occurs through the blockade of the CNP cascade. Future studies will need to address changes in the protein levels and activities in response to glucocorticoids to assess whether the observed changes in transcripts indeed result in increased CNP signaling. However, recent studies using transgenic mice show that increased CNP mRNA levels directly cause increased CNP signalling [19,27], suggesting that induction of endogenous CNP mRNA (e.g. in response to DEX) results in increased production of active CNP in a similar manner.

Our data also show that DEX induces modest down-regulation in the expression of its own receptor. These data suggest the existence of a negative feedback response loop limiting glucocorticoid effects, a scenario that is consistent with earlier reports demonstrating that DEX down-regulates expression of the glucocorticoid receptor in other cell types [39]. Nevertheless, cells remained responsive to DEX as demonstrated by our gene expression profiles. In this context, it is of interest that among all examined genes, only *Nppc *and *Prkg2 *responded to DEX at the earliest tested time point (12 hours). These data suggest that *Nppc *could be a direct target of transcriptional regulation by glucocorticoids. However, sequence analyses of the mouse *Nppc *gene did not identify any apparent glucocorticoid response elements (data not shown). In contrast to *Nppc *and *Prkg2*, all other examined genes displayed a slower response to DEX treatment and are likely controlled by glucocorticoids through indirect mechanisms. For example, it has been shown earlier that CNP suppresses the expression of its own receptor, NPR2 [40]. The observed decrease in *Npr2 *mRNA levels in response to DEX might therefore be secondary to increased CNP levels. Similarly, our recent studies show that increased CNP levels promote the expression of the decoy receptor encoded by *Npr3*, in another negative feedback loop (Agoston et al., submitted). However, an alternative explanation is that other target genes of glucocorticoids (outside of the CNP signalling system) are responsible for the changes in *Npr2 *and *Npr3 *expression.

These studies with primary cells provide the impetus for future exploration into the interactive roles of the glucocorticoid and natriuretic peptide pathways. Despite the opposing roles of the glucocorticoid and CNP signaling pathways in endochondral bone growth, the strong upregulation of CNP mRNA expression by DEX is intriguing, and the elucidation of its role in the cartilage response to glucocorticoids will be of great interest. Furthermore, these studies also demonstrate that the endogenous expression of CNP (and some of the genes mediating CNP signaling) during skeletal development is controlled by endocrine factors.

## Conclusion

Our data show that DEX regulates the expression of several components of the CNP signaling pathway. Therefore, our study identifies a novel and potentially important interaction between two pathways that both control endochondral ossification and that both have been implicated in pathologies of endochondral bone growth. Further studies into these pathways and their interactions will contribute to a deeper understanding of endochondral ossification and associated pathologies.

## Abbreviations

cGK, cyclic GMP-dependent kinase; CNP, C-type natriuretic peptide; DEX, dexamethasone; NPR, natriuretic peptide receptor; PCR, polymerase chain reaction

## Competing interests

The author(s) declare that they have no competing interests.

## Authors' contributions

HA performed the real-time PCR analyses, co-wrote the manuscript and contributed to the design of the study. LB carried out the proliferation studies. FB conceived and designed the study and co-wrote the manuscript. All authors read and approved the final manuscript.

## Pre-publication history

The pre-publication history for this paper can be accessed here:
